# Maternal and Newborn Thyroid Hormone, and the Association With Polychlorinated Biphenyls (PCBs) Burden: The EHF (Environmental Health Fund) Birth Cohort

**DOI:** 10.3389/fped.2021.705395

**Published:** 2021-09-13

**Authors:** Maya Berlin, Dana Barchel, Anna Brik, Elkana Kohn, Ayelet Livne, Rimona Keidar, Josef Tovbin, Moshe Betser, Miki Moskovich, Dror Mandel, Ronit Lubetzky, Amit Ovental, Pam Factor-Litvak, Malka Britzi, Tomer Ziv-Baran, Ronit Koren, Chagit Klieger, Matitiahu Berkovitch, Ilan Matok, Ronella Marom

**Affiliations:** ^1^Clinical Pharmacology and Toxicology Unit, Shamir Medical Center (Assaf Harofeh), Affiliated to Sackler Faculty of Medicine, Tel-Aviv University, Tel Aviv, Israel; ^2^Division of Clinical Pharmacy, Institute for Drug Research, School of Pharmacy, Faculty of Medicine, Hebrew University of Jerusalem, Jerusalem, Israel; ^3^Department of Neonatology, Shamir Medical Center (Assaf Harofeh), Affiliated to Sackler Faculty of Medicine, Tel-Aviv University, Tel Aviv, Israel; ^4^Division of Obstetrics and Gynecology, Shamir Medical Center (Assaf Harofeh), Affiliated to Sackler Faculty of Medicine, Tel-Aviv University, Tel Aviv, Israel; ^5^Departments of Neonatology and Pediatrics, Dana Dwek Children's Hospital, Tel Aviv Medical Center, Affiliated to Sackler Faculty of Medicine, Tel-Aviv University, Tel Aviv, Israel; ^6^Department of Epidemiology, Mailman School of Public Health, Columbia University, New York, NY, United States; ^7^Residues Lab, Kimron Veterinary Institute, Beit-Dagan, Israel; ^8^Department of Epidemiology and Preventive Medicine, School of Public Health, Sackler Faculty of Medicine, Tel-Aviv University, Tel Aviv, Israel; ^9^Department of Internal Medicine A, Shamir Medical Center (Assaf Harofeh), Affiliated to Sackler Faculty of Medicine, Tel-Aviv University, Tel Aviv, Israel; ^10^Feto-Maternal Unit, Lis Hospital, Tel Aviv Medical Center, Affiliated to Sackler Faculty of Medicine, Tel-Aviv University, Tel Aviv, Israel

**Keywords:** polychlorinated biphenyls (PCBs), endocrine-disrupting chemicals (EDCs), thyroid hormones, pregnancy, intrauterine exposure

## Abstract

**Background:** Polychlorinated biphenyls (PCBs) are ubiquitous environmental contaminants found in human tissues. PCBs can be transferred through the placenta and may disrupt the maternal thyroid homeostasis, and affect fetal thyroid hormone production. Several studies have shown that intrauterine exposure to PCBs might be associated with abnormal levels of thyroid hormones in mothers and their offspring.

**Objectives:** To examine the associations between environmental exposure to PCBs and thyroid hormone levels in mothers and newborns.

**Methods:** The EHF-Assaf-Harofeh-Ichilov cohort includes 263 mothers-newborns dyads. A total of 157 mother-newborn dyads had both PCBs and thyroid function measures. Regression models were used to estimate associations between maternal PCB exposure and maternal and newborn thyroid function, controlling for possible confounders.

**Results:** Four PCBs congeners were analyzed: PCBs 118, 138, 153, and 180. ∑PCBs median (IQR) level was 14.65 (2.83–68.14) ng/g lipids. The median maternal thyroid-stimulating hormone (TSH) level was 2.66 (0.70–8.23) μIU/ml, the median maternal free thyroxine (FT4) level was 12.44 (11.27–13.53) μg/dL, the median maternal thyroid peroxidase antibodies (TPO Ab) level was 9.6 (7.36–12.51) IU/mL. Newborns' median total thyroxine (T4) level was 14.8 (7.6–24.9) μg/dL. No association was found between exposure to different congeners or to ∑PCBs and maternal TSH, FT4, thyroglobulin autoantibodies (Tg Ab), TPO Ab and newborn total T4 levels. In multivariable analysis a 1% change in ∑PCBs level was significantly associated with a 0.57% change in maternal TSH levels in women with body mass index (BMI) < 19. The same association was observed for each of the studied PCB congeners. Maternal TPO Ab levels statistically significantly increased by 0.53 and 0.46% for 1% increase in PCB 118 and 153 congeners, respectively. In women with BMI > 25, the association between the PCBs levels and maternal TSH levels was in the opposite direction. No association was found in women with normal BMI (19–24.9).

**Conclusions:** Background exposure to environmentally relevant concentrations of some PCBs can alter thyroid hormone homeostasis in pregnant women and might be associated with abnormal TSH levels and TPO-Ab in women with low BMI. However, these findings require further investigation.

## Introduction

Endocrine-disrupting chemicals (EDCs) contribute substantially to human morbidity and are estimated to result in hundreds of billions in costs per year (1). The Endocrine Society presented evidence that EDCs have multiple effects on multiple endocrine systems, including those associated with female reproduction, thyroid function, neurodevelopment, and neuroendocrine systems especially during development (2).

Polychlorinated biphenyls (PCBs) are a large family of persistent endocrine-disrupting industrial chemicals, once widely used as non-flammable lubricants and insulators (3, 4). PCBs were banned from production in the late 1970s in the USA and from 2001 onwards worldwide by the Stockholm Convention on Persistent Organic Pollutants (5). However, their chemical nature, lipophilicity, and resistance to degradation have led to bioaccumulation in the ecosystem, with ongoing human exposure (6). Due to their lipophilic structure, PCBs are readily absorbed from the environment into the food chain, rendering human and animal exposure ubiquitous. Human exposure mainly involves consuming fatty food like fish, meat, and dairy products (7, 8). PCBs 118, 138, 153, 180 are among the most frequently detected congeners in white adipose tissue and blood samples (9–11). These PCBs are more heavily chlorinated and have a half-life of years (12). PCBs 138, 153, and 180 have the highest detection frequencies in the US population and contributed to 80% of the total PCBs in human serum (13). PCB 118 is considered a dioxin-like compound, mono-*ortho* PCB, where PCBs 138, 153, and 180 are non-dioxin-like congeners, di-*ortho* congeners (3, 4).

Some studies examined PCBs as endocrine disruptors both in animals (14, 15) and in humans, with a particular focus on thyroid hormones (16, 17) and steroid hormones related to the reproductive system (18). In particular, associations between PCB serum levels and thyroid function have been examined both in adults (19) where thyroid hormone differences were observed in relation to serum PCB concentrations, and neonates (20) showing that prenatal exposure to some PCBs impacts newborn TSH levels.

Thyroid function is considered a potential target for endocrine disruption by persistent chemicals such as PCBs (16, 17, 21). PCBs may disrupt thyroid hormone signaling and lead to thyroid disease given the structural similarities to thyroid hormones (2). In particular, PCBs can disrupt estrogen receptors, aryl hydrocarbon receptors (AhR), or thyroid hormone receptors (22, 23). Thangavelu et al. suggest that PCBs exposure may reduce the serum levels of LH, testosterone and estradiol, may cause a decrease in the Leydig cell population and may decrease activities of steroidogenic enzymes 3β- and 17β-HSD (24).

A recent study showed that serum AhR bioactivities were increased with specific PCB congeners, regardless of their dioxin-like properties (25). Miyazaki et al. posited that weak suppression of TR-mediated transcription by non-dioxin-like PCBs is caused by dissociating thyroid hormone receptor (TR) from the TR response element (TRE) (26). The combined data from human studies and experimental animal studies also suggest that PCBs negatively affect thyroid function by interference with transport and metabolism (27). Curtis et al. found that higher Polybrominated biphenyls (PBBs) were associated with higher free T3, lower free T4, and a higher FT3/FT4 ratio and higher PCBs levels were associated with higher FT4 and a higher FT3/FT4 ratio (28).

It is known that adequate levels of thyroid hormones are crucial for the proper development of the brain. Epidemiological studies found that subclinical disruptions in thyroid hormones during pregnancy are associated with decrements in cognitive functions of offspring later in life. Hence, exposure to thyroid-disrupting chemicals may have significant consequences for public health even if they cause a small change in thyroid hormone levels. This could explain some, but not all of the neurodevelopmental effects of PCBs; however, data are not consistent across the studies (2, 11, 29–34).

Pregnant women, fetuses, infants, and children are most vulnerable to low-dose environmental exposures. There is growing evidence of adverse effects of environmental exposure on reproduction, pre and postnatal development (35–37). As a lipophilic substance, PCBs can be transferred through the placenta and during breastfeeding to increase children's body burden. Therefore, children are exposed to PCBs starting from conception, and the exposure continues throughout different stages of their lives (38). Exposure to these chemicals may have adverse effects on the developing fetus, including intrauterine growth retardation (IUGR), neurocognitive deficit, and hormonal dysfunction (39–41). Organochlorines, including PCBs, have been shown to cross the placenta and have been associated with neurobehavioral effects in children (42, 43).

Some studies suggest that TSH values differ in pregnant women as pre-pregnancy body mass index (BMI) increases, and there is an association between lower FT4 levels and higher BMI (44). In addition, there are differences in body fat distribution between overweight and normal-weight women during pregnancy (45). Changes in maternal fat mass can range from −10 to +15 kg at term (46).

In this study we aimed to evaluate the possible associations between background exposure to PCBs and thyroid hormone levels within a birth cohort of Israeli mothers and newborns.

## Materials and Methods

### Study Population

We used data collected as part of a birth cohort performed at Shamir Medical Center (Assaf Harofe) and “Dana Dwek” Children's Hospital, Tel Aviv Medical Center.

From January 2013 through April 2015, 263 mother-father-newborn triads were recruited. The women recruited into the study were asked to participate during attendance in the delivery room. Data on social and demographic characteristics and lifestyle variables from both the father and mother were obtained through a detailed questionnaire. Data on occupation, residential history, diet, hobbies, and a detailed health history were also collected. At the delivery room blood and urine samples were collected from mothers and fathers. After delivery, cord blood samples, meconium samples, and breast milk (colostrum) samples were collected. All samples were processed according to a standard protocol, and frozen at −80°C.

Maternal height, pre-pregnancy weight, and weight at the time of delivery were extracted from medical records. As part of the routine physical examination, all infants underwent a structured examination for congenital malformations.

Birth weight, length, and head circumference were measured three times using standard research procedures.

To compare the birth weights, birth weight was adjusted to gestational age at birth and infant's sex and classified according to percentile values derived from the Israeli Perinatal survey (47).

All participants signed informed consent. The study was performed according to the Declaration of Helsinki, and the Institutional Review Board approved the protocol (48).

### Data Extraction

The database included 263 mother-newborn dyads. Due to financial limitations, we measured concentrations of 4 PCBs from maternal blood of 183 mothers who gave birth at Shamir Medical Center (Assaf Harofeh). On average, included and excluded dyads were similar in terms of potential confounders such as birth weight, length of gestation, and maternal age (49).

The comparison of maternal characteristics of included and excluded dyads is presented in Supplementary Table 1. There were more primiparous included in the study (31.8 vs. 18.9%, *p* = 0.015). Pre-pregnancy BMI was lower in the included mothers compared to the excluded (median, IQR), 21.1 (19.3–22.9) vs. 22.5 (20.5–24.3), *p* = 0.008. Excluded mothers had a higher percentage of Foreign-born (27 vs. 15.3%, *p* = 0.022).

We retrieved data on 183 dyads for which maternal serum PCBs measurements were obtained. Data on maternal demographics, exposures, lifestyle, and characteristics of labor were extracted as well as newborn anthropometrics and measurements.

Maternal laboratory test results, obtained at the time of delivery, included thyroid stimulating hormone (TSH), free thyroxine (FT4), thyroglobulin autoantibodies (TgAb), thyroid peroxidase antibody test (TPOAb), total cholesterol, and triglycerides.

Newborn total T4 was obtained through a routine national survey methodology testing for congenital hypothyroidism by heel puncture blood sample, collected between days 2–3 of life.

Cases of women with known hypothyroidism (*N* = 9) or hyperthyroidism (*N* = 2), dyads with no total T4 levels for the newborn (*N* = 12), twin pregnancies (*N* = 2), and premature delivery (<35 weeks) (*N* = 1) were excluded.

### Sample Analysis

PCBs were measured in 200 randomly selected maternal samples. Seventeen of the 200 samples were duplicates. The correlations between the duplicates were 0.996, 0.997, 1, and 0.997 for congeners 118, 138, 153, and 180, respectively. Where duplicate samples were measured, a mean of the two results was taken.

Blood samples were collected through venipuncture into tubes containing clot activator. Samples were kept at 4°C until processing, within 4–6 h from collection. Samples were centrifuged at 1,200 g for 7 min and serum was aliquoted in 2 ml cryovials and kept frozen at −80°C until analysis.

PCBs were measured at the Centre de Toxicologie du Quebec. Congeners 118 (2,3′,4,4′,5-pentachlorobiphenyl), 138 (2,2′,3,4,4′,5′-hexachlorobiphenyl), 153 (2,2′,4,4′,5,5′-hexachlorobiphenyl), and 180 (2,2′,3,4,4′,5,5′-heptachlorobiphenyl) were measured. All measures were performed using GC/MS at INSPEQ, at the Arctic Monitoring and Assessment Programme Ring Test for Persistent Organic Pollutants in Human Serum (AMAP), organized and managed by the Centre de Toxicologie du Québec (CTQ).

The lower limit of quantification (LOQ) was 10 ng/L. For statistical analysis purposes, values below LOQ were assigned with the value of LOQ/√(2). All the samples were above the LOQ for the PCB 153. Additionally, there were 11 samples with levels below the LOQ: PCB 118—eight samples, PCB 118+138—one sample, PCB 118+180—one sample, PCB 118+138+180—one sample.

PCBs were reported as the wet weight. We normalized the concentrations using the following equation which adjusts chemical per gram of total lipids (lipid weight, ng chemical/g total lipid) (50).

Total lipids (TL) were estimated using the formula (51, 52):

TL (g/L) = 2.27 × TC (g/L) + TG (g/L) + 0.623

where TL—total lipids, TC—total cholesterol, TG—triglycerides.

Maternal TSH, FT4, thyroglobulin autoantibodies (TgAb), thyroid peroxidase antibody test (TPOAb), total cholesterol, and triglycerides were measured at the Shamir (Assaf Harofeh) biochemistry lab using standard methods.

The enzymatic method was used for the quantitative determination of cholesterol and triglycerides in human serum and plasma on Roche/Hitachi Cobas c systems. Immunoassay was used for the quantitative determination of free T4, TSH, TPOAb, and TgAb.

TPO Ab was defined positive above 34 IU/mL, and Tg Ab was defined positive above 115 IU/mL.

### Statistical Analysis

Continuous variables are presented as mean and standard deviation (±SD) or median and interquartile range (IQR). Continuous variables were compared between groups using the Kruskal Wallis test or Mann-Whitney test. Categorical variables were compared using the Chi-square test, or Fisher's exact test, as appropriate.

Spearman correlation coefficients were calculated to assess the crude correlation between thyroid function of the mother and the newborn and PCBs level variables.

Potential confounders were selected based on their previously reported associations with prenatal PCB exposure or with thyroid function. Potential confounders included: gender, country of origin, date of birth, gestational age (in weeks), birth weight (in grams), prenatal tobacco smoke exposure, parity (nulliparous/multiparous), relationship status (unmarried/married or with the same partner), maternal age (in years), monthly household income (net), maternal education, and maternal employment. Our final models adjusted for maternal age and newborn sex since the association between males and females and the total T4 in newborns was different.

The maternal PCBs levels, TSH levels, FT4, TPOAb, TgAb, and newborn total T4 were skewed; therefore, we used natural logarithm transformation to achieve normality. Linear regression analysis was used to examine the associations between the exposure variables and the outcome variables, adjusting for potential confounding variables.

Since natural logarithm transformation was used for the dependent and independent variables, the regression describes the percent change in TSH levels according to a 1 percent change in the PCB levels.

Interaction variables between pre-pregnancy BMI categories and PCB levels and between newborn sex and PCB levels were included in the model (53).

All statistical tests were two-tailed, and *p* < 0.05 was considered statistically significant. SPSS software (IBMS SPSS Statistics for Windows, Version 25, IBM Corp, Armonk, New York, USA) was used for all statistical analyses.

## Results

One hundred and fifty-seven mothers-newborn dyads were included in the final analysis. The mothers' characteristics including age, education, relationship, employment status, monthly income, alcohol and smoking habits, and country of origin are presented in Table 1. The data presented in Tables 1–**3** are grouped by maternal pre-pregnancy BMI.

**Table 1:** Demographic characteristics of mothers in the EHF-Assaf-Harofeh-Ichilov birth cohort, Israel, 2013–2015, grouped by maternal pre-pregnancy BMI.

**Maternal characteristics**	**ALL**	**BMI <19**	**BMI = 19–25**	**BMI ≥ 25**	***P***
**Number**	**157**	**24**	**88**	**45**	
Maternal age at delivery (years) Mean ± SD	31.9 ± 4.8	30.96 ± 5.1^*^	30.3 ± 4.5^#^	33.6 ± 5.1^*^^#^	0.03^*^^#^
**Parity**					
Primiparous (yes)	50 (31.8%)	7 (29.2%)	34 (38.6%)	9 (20%)	0.09
**Education/Social/Habits**					0.84
High school or less (*N*, %)	29 (18.5%)	5 (20.8%)	18 (20.5%)	6 (13.3%)	
College or trade school diploma (*N*, %)	29 (18.5%)	4 (16.7%)	15 (17.0%)	10 (22.2%)	
Bachelor or higher (*N*, %)	98 (62.4%)	15 (62.5%)	54 (61.4%)	29 (64.4%)	
Stable relationship (yes) (*N*, %)	152 (96.8%)	24 (100%)	85 (96.6%)	43 (95.6%)	0.84
Employed (yes) (*N*, %)	138 (87.9%)	22 (91.7%)	77 (87.5%)	39 (86.7%)	0.47
Has ever smoked during pregnancy (yes) (*N*, %)	11 (7.0%)	1 (4.2%)	6 (6.8%)	4 (8.9%)	0.83
Has ever consumed alcohol during pregnancy (yes) (*N*, %)	13 (8.3%)	2 (8.3%)	8 (9.1%)	3 (6.7%)	0.92
**Monthly Household Income (Net)**					0.31
<17,000 NIS (yes) (*N*, %)	106 (67.5%)	14 (58.3%)	58 (65.9%)	34 (75.6%)	
17,001 NIS and more (yes) (*N*, %)	38 (24.2%)	8 (33.3%)	22 (25%)	8 (17.8%)	
**Country of origin**					0.57
Israel (*N*, %)	133 (84.7%)	22 (91.7%)	74 (84.1%)	37 (82.2%)	
Other (*N*, %)	24 (15.3%)	2 (8.3%)	14 (15.9%)	8 (17.8%)	
Maternal pregnancy weight gain (kg) median, IQR	13 (10–16)	13.5 (10.25–18)	13 (10–16)	12.5 (7.75–16.25)	0.28

*,#: *groups with statistically significant difference*.

*IQR, interquartile range; TSH, Thyroid-stimulating hormone; FT4, Free Thyroxine; PCB, polychlorinated biphenyl; SD, Standard Deviation*.

*Kruskal Wallis, Pearson Chi-square or Fisher's exact tests were used*.

*Bold: p-value < 0.05*.

The maternal mean (±SD) age was 31.9 ± 4.8 years. Women with higher pre-pregnancy BMI were older that other 2 groups: 33.6 ± 5.1 years in BMI ≥ 25, 30.96 ± 5.1 years and 30.3 ± 4.5 years in BMI < 19 and BMI 19–24.9 respectively (*p* = 0.03). No other differences were observed between the 3 BMI groups. About one-third of women were primiparous (31.8%). Most women had an academic education −98 (62.4%) with a bachelor's degree or higher. Almost all women were married (96.8%) and employed (87.9%). One hundred and thirty-three mothers were born in Israel (84.7%), and most foreign-born mothers (21/24, 87.5%) were born in East Europe. Only a few women consumed alcohol or smoked during pregnancy (*N* = 13, 8.3% and *N* = 11, 7.0%, respectively).

Maternal PCBs' concentration levels (lipid adjusted) are presented in Table 2. PCBs 118, 138, 153, and 180 median levels were 2.23 (1.79–3.03), 3.31 (2.51–4.76), 5.44 (3.83–7.67), and 3.69 (2.67–5.4) ng/g lipids, respectively. ∑PCBs median level was 14.65 (11.03–20.89) ng/g lipids. No statistically significant differences were observed between the 3 BMI groups. Total lipids were calculated as described in the Material and Methods section (Sample Analysis) and no statistically significant differences were observed between the three BMI groups (*p* = 0.06) (Table 2).

**Table 2:** Maternal measurements in the EHF-Assaf-Harofeh-Ichilov birth cohort Israel, 2013–2015, grouped by maternal pre-pregnancy BMI.

	**ALL**	**BMI <19**	**BMI = 19–25**	**BMI ≥ 25**	***P***
**Number**	**157**	**24**	**88**	**45**	
PCB 118 (ng/g lipids) (median, IQR)	2.23 (1.79–3.03)	2.05 (1.64–2.59)	2.22 (1.75–3.04)	2.53 (1.9–3.56)	0.13
PCB 138 (ng/g lipids) (median, IQR)	3.31 (2.51–4.76)	3.26 (2.45–4.48)	3.27 (2.53–4.52)	3.94 (2.48–5.5)	0.38
PCB 153 (ng/g lipids) (median, IQR)	5.44 (3.83–7.67)	5.32 (3.64–9.72)	5.17 (3.87–6.85)	6.06 (3.99–8.67)	0.34
PCB 180 (ng/g lipids) (median, IQR)	3.69 (2.67–5.4)	3.9 (2.67–6.18)	3.53 (2.57–4.82)	4.26 (2.74–7.09)	0.22
Sum PCBs (ng/g lipids) (median, IQR)	14.65 (11.03–20.89)	14.78 (10.03–22.67)	14.09 (11.14–18.73)	17.07 (10.94–23.11)	0.22
TSH μIU/ml (median, IQR)	2.66 (2.0–3.54)	2.79 (2.49–4.24)	2.65 (2.07–3.47)	2.55 (1.95–3.37)	0.37
Maternal FT4 μg/dl (median, IQR)	12.44 (11.27–13.53)	12.95 (11.49–14.52)	12.54 (11.84–13.57)	11.55 (10.62–13.06)	0.08
Tg Ab IU/mL (median, IQR)	13.51 (10.88–16.84)	14.63 (11.32–18.7)	13.44 (10.59–16.43)	13.31 (10.95–16.45)	0.59
TPO Ab IU/mL (median, IQR)	9.6 (7.36–12.51)	10.36 (7.88–16.01)	9.89 (6.9–12.21)	8.99 (7.63–12.43)	0.41
Total lipids g/L (median, IQR)	9.01 (7.99–10.43)	9.78 (7.88–11.13)	9.11 (8.02–10.6)	8.7 (7.72–9.88)	0.06

*IQR, interquartile range; TSH, Thyroid-stimulating hormone; FT4, Free Thyroxine; PCB, polychlorinated biphenyl; Tg Ab, Thyroglobulin autoantibodies; TPO Ab, Thyroid peroxidase antibodies; TG, triglycerides.*

*Kruskal Wallis, Pearson Chi-square or Fisher's exact tests were used*.

*Bold: p-value < 0.05*.

Maternal median FT4 and TSH levels were 12.44 (IQR 11.27–13.53, laboratory range 12–22) μg/dl and 2.66 (IQR 2.0–3.54, laboratory range 0.27–4.2) μIU/ml, respectively. Maternal Tg Ab and TPO Ab were measured 13.51 (IQR 10.88–16.84) IU/mL and 9.6 (IQR 7.36–12.51) IU/mL, respectively. Three women had thyroid peroxidase antibodies >34 IU/L and were categorized as anti-TPO positive, one in each BMI group (Table 2). No statistically significant differences were observed between the 3 BMI groups.

Newborn mean (±SD) birth week was 39.33 ± 1.28. The newborn mean (±SD) birth weight was 3,141 g (±474.25). Newborn mean (±SD) birth length was 49.28 cm (±2.08). Newborns of women with higher BMI had higher birth weight, birth length and head circumference than the other 2 BMI groups: 3,236 g (±454.87), 49.86 cm (±2.06), 34.27 cm (±1.31), (*p*-value: 0.02, 0.03, 0.049), respectively (Table 3).

**Table 3:** Anthropometric measurements and characteristics of the newborns in the EHF-Assaf-Harofeh-Ichilov birth cohort, Israel, 2013–2015, grouped by maternal pre-pregnancy BMI.

	**ALL**	**BMI <19**	**BMI = 19–25**	**BMI ≥ 25**	***P***
**Number**	**157**	**24**	**88**	**45**	
Male (*N*, %)	85 (54.1%)	10 (41.7%)	49 (55.7%)	26 (57.8%)	0.4
Gestational age (weeks) Mean ± SD	39.33 ± 1.28	38.88 ± 1.26	39.37 ± 1.27	39.49 ± 1.29	0.34
Birth weight (g) Mean ± SD	3,141 474.25	2,894 407.53^*^^#^	3,147 467.23^*^	3,236 454.87^#^	0.02^*^#
Birth length (cm) Mean ± SD	49.28 ± 2.08	48.47 ± 2.03^*^	49.22 ± 2.04	49.86 ± 2.06^*^	0.03^*^
Head circumference (cm) Mean ± SD	34.09 ± 1.21	33.47 ± 0.99^*^	34.17 ± 1.17^*^	34.27 ± 1.31	0.049^*^
Newborn weight percentile^a^ (median, IQR)	45 (22.5–66.5)	32 (12.5–49.75)	45 (23.75–65.75)	58 (21.5–77)	0.057
NB T4 μg/dl (median, IQR)	14.8 (12.8–17.05)	15.05 (13.73–17.27)	12.53 (11.83–13.57)	11.55 (10.61–13.06)	0.66
**Delivery method**					0.17
Vaginal (*N*, %)	94 (59.9%)	15 (62.5%)	57 (64.8%)	22 (48.9%)	
C/S (*N*, %)	63 (40.1%)	9 (37.5%)	31 (35.2%)	23 (51.1%)	

a *By Dollberg et al. (47)*.

*,#: *groups with statistically significant difference*.

*IQR, interquartile range; C/S, cesarean section delivery; NB T4, newborn total thyroxine; SD, Standard Deviation*.

*Kruskal Wallis or Pearson Chi-square tests were used*.

*Bold: p-value < 0.05*.

Newborn weight percentile was higher in women with BMI ≥ 25, as compared with the other 2 BMI groups, median (IQR) −58 (21.5–77), vs. 32 (12.5–49.75) and 45 (23.75–65.75), *p* = 0.057. No other differences were observed between the 3 BMI groups.

Vaginal delivery method was recorded in 59.9% (94) of cases. Newborn median total T4 level was 14.8 μg/dL (IQR 12.8–17.05). The ranges of newborn total T4 levels were within what is considered a standard reference range (Table 3).

No correlation was found between maternal exposure to the studied PCB congeners or ∑PCBs and maternal TSH, maternal FT4, maternal TG Ab, maternal TPO Ab, and total T4 levels in the newborns in the total study population (Table 4).

**Table 4:** Spearman rank correlations of PCBs (lipid adj.) and thyroid function of mothers and newborns in the EHF-Assaf-Harofeh-Ichilov birth cohort, Israel, 2013–2015 (*n* = 157).

		**Maternal TSH**	**Maternal FT4**	**Maternal**	**Maternal**	**NB T4**
				**Tg Ab**	**TPO Ab**	
PCB 118 (ng/g lipids)	r	−0.019	0.021	0.029	−0.015	0.131
	p	0.814	0.798	0.717	0.849	0.102
PCB 138 (ng/g lipids)	r	−0.022	0.003	0.099	0.083	0.118
	p	0.789	0.975	0.217	0.299	0.142
PCB 153 (ng/g lipids)	r	−0.028	0.030	0.102	0.084	0.100
	p	0.730	0.710	0.204	0.296	0.212
PCB 180 (ng/g lipids)	r	−0.045	0.031	0.081	0.055	0.043
	p	0.573	0.704	0.311	0.493	0.597
Sum PCBs (ng/g lipids)	r	−0.024	0.025	0.090	0.064	0.093
	p	0.763	0.760	0.260	0.428	0.249
**Male (** ***N*** **= 85)**						
PCB 118 (ng/g lipids)	r	−0.074	0.038	0.077	−0.075	**0.250**
	p	0.501	0.730	0.481	0.495	**0.021^#^**
PCB 138 (ng/g lipids)	r	−0.101	0.093	0.152	0.005	**0.247**
	p	0.360	0.397	0.166	0.963	**0.022^#^**
PCB 153 (ng/g lipids)	r	−0.096	0.103	0.176	0.031	**0.232**
	p	0.382	0.347	0.107	0.781	**0.032^#^**
PCB 180 (ng/g lipids)	r	−0.083	0.101	0.185	0.042	0.194
	p	0.450	0.359	0.090	0.705	0.076
Sum PCBs (ng/g lipids)	r	−0.080	0.099	0.164	0.015	**0.225**
	p	0.465	0.369	0.133	0.894	**0.039^#^**
**Female (** ***N*** **= 72)**						
PCB 118 (ng/g lipids)	r	0.047	0.009	−0.004	0.053	−0.066
	p	0.693	0.938	0.972	0.661	0.582
PCB 138 (ng/g lipids)	r	0.072	−0.082	0.056	0.180	−0.063
	p	0.548	0.496	0.640	0.129	0.600
PCB 153 (ng/g lipids)	r	0.040	−0.022	0.040	0.144	−0.084
	p	0.737	0.852	0.740	0.229	0.481
PCB 180 (ng/g lipids)	r	0.016	−0.025	0.000	0.065	−0.170
	p	0.894	0.833	0.997	0.588	0.153
Sum PCBs (ng/g lipids)	r	0.040	−0.025	0.029	0.126	−0.089
	p	0.739	0.838	0.810	0.293	0.457

*r, Spearman's rho*.

*p, p-value*.

# *P < 0.05*.

*TSH, Thyroid-stimulating hormone; FT4, Free Thyroxine; Tg Ab, Thyroglobulin autoantibodies; TPO Ab, Thyroid peroxidase antibodies; NB T4, newborn total thyroxine; PCB, polychlorinated biphenyl*.

*Bold: p-value < 0.05*.

A statistically significant correlation was found between maternal PCBs and total T4 in male, but not in female neonates. Spearman rank correlations for males' r (*p*-value): 0.250 (0.021), 0.247 (0.022), 0.232 (0.032), and 0.194 (0.076) for PCBs 118, 138, 153, and 180, respectively, and 0.225 (0.039) for ∑PCBs. Spearman rank correlations for females' r (*p*-value): −0.066 (0.582), −0.063 (0.6), −0.084 (0.481), −0.170 (0.153), and −0.089 (0.457) for PCBs 118, 138, 153, 180, and for ∑PCBs, respectively (Table 4).

Since a significant interaction between maternal pre-pregnancy BMI and maternal PCBs levels was found, the association between thyroid functions and PCBs was studied separately in each BMI group.

All 4 studied PCB congeners and ∑PCBs were highly correlated to maternal TSH in BMI < 19 group. Spearman rank correlations between PCB 118, 138, 153, 180, ∑PCBs and maternal TSH levels were 0.412 (*p* value: 0.045), 0.501 (*p* value: 0.013), 0.518 (*p* value: 0.010), 0.445 (*p* value: 0.03), and 0.511 (*p* value: 0.011), respectively (Table 5).

**Table 5:** Spearman rank correlations of PCBs (lipid adj.) and thyroid function of mothers and newborns at different BMI groups, in the EHF-Assaf-Harofeh-Ichilov birth cohort, Israel, 2013–2015, grouped by maternal pre-pregnancy BMI.

		**Maternal TSH**	**Maternal FT4**	**Maternal**	**Maternal**	**NB T4**
				**Tg Ab**	**TPO Ab**	
**BMI <19 (** ***N*** **= 24)**						
PCB 118 (ng/g lipids)	r	**0.412**	0.287	0.341	**0.477**	−0.237
	p	**0.045^#^**	0.175	0.103	**0.018^#^**	0.265
PCB 138 (ng/g lipids)	r	**0.501**	0.358	0.191	**0.437**	−0.139
	p	**0.013^#^**	0.086	0.370	**0.033^#^**	0.517
PCB 153 (ng/g lipids)	r	**0.518**	0.305	0.178	0.401	−0.177
	p	**0.010^#^**	0.147	0.406	0.052	0.408
PCB 180 (ng/g lipids)	r	**0.445**	0.160	0.048	0.277	−0.215
	p	**0.030^#^**	0.454	0.823	0.191	0.313
Sum PCBs (ng/g lipids)	r	**0.511**	0.262	0.150	0.376	−0.141
	p	**0.011^#^**	0.216	0.483	0.070	0.510
**BMI 19–25 (** ***N*** **= 88)**						
PCB 118 (ng/g lipids)	r	0.033	−0.019	−0.066	−0.208	0.160
	p	0.760	0.860	0.542	0.052	0.136
PCB 138 (ng/g lipids)	r	0.002	−0.038	0.048	−0.057	0.192
	p	0.984	0.724	0.654	0.596	0.073
PCB 153 (ng/g lipids)	r	−0.025	0.009	0.098	−0.022	0.147
	p	0.820	0.934	0.363	0.842	0.170
PCB 180 (ng/g lipids)	r	−0.019	0.034	0.112	−0.021	0.076
	p	0.858	0.752	0.298	0.849	0.483
Sum PCBs (ng/g lipids)	r	−0.009	0.009	0.073	−0.052	0.146
	p	0.933	0.936	0.496	0.629	0.176
**BMI ≥ 25 (** ***N*** **= 45)**						
PCB 118 (ng/g lipids)	r	−0.224	0.079	0.134	0.179	0.275
	p	0.139	0.607	0.380	0.240	0.068
PCB 138 (ng/g lipids)	r	−0.255	0.010	0.139	0.177	0.148
	p	0.091	0.947	0.361	0.245	0.331
PCB 153 (ng/g lipids)	r	−0.265	0.039	0.088	0.116	0.182
	p	0.079	0.800	0.567	0.449	0.232
PCB 180 (ng/g lipids)	r	−0.290	0.048	0.052	0.085	0.157
	p	0.053	0.754	0.732	0.579	0.302
Sum PCBs (ng/g lipids)	r	−0.276	0.051	0.095	0.126	0.192
	p	0.066	0.738	0.535	0.411	0.206

*r, Spearman's rho*.

*p, p-value*.

# *p < 0.05*.

*TSH, Thyroid-stimulating hormone; FT4, Free Thyroxine; Tg Ab, Thyroglobulin autoantibodies; TPO Ab, Thyroid peroxidase antibodies; NB T4, newborn total thyroxine; PCB, polychlorinated biphenyl*.

*Bold: p-value < 0.05*.

Maternal TPO Ab were highly correlated to congeners PCB 118 and 138, and approached significance for PCB 153 but not for 180 or ∑PCBs. Spearman rank correlations between PCB 118, 138, 153, 180, ∑PCBs and maternal TPO Ab were 0.477 (*p* value: 0.018), 0.437 (*p* value: 0.033), 0.401 (*p* value: 0.052), 0.277 (*p* value: 0.191), and 0.376 (*p* value: 0.070), respectively (Table 5).

No correlation was found between maternal FT4, maternal Tg Ab, and newborn total T4 and the studied PCB congeners or ∑PCBs within different BMI groups (Table 5).

After adjustment, in women with pre-pregnancy BMI < 19 group, a 1% increase in the ∑PCBs level was associated with a 0.57% increase in the maternal TSH levels and a 0.47, 0.59, 0.51, and 0.44% increase for a 1% increase in the PCBs 118, 138, 153, and 180, respectively. Maternal TPO Ab levels were statistically significantly increased by 0.53 and 0.46% for a 1% increase in PCB 118 and 153, respectively (Table 6).

**Table 6:** Adjusted^§^ association between maternal PCB concentrations (lipid adj.) to thyroid function of mothers and newborns in the EHF-Assaf-Harofeh-Ichilov birth cohort, Israel, 2013–2015, grouped by maternal pre-pregnancy BMI (*n* = 157).

	**PCB 118 (95% CI)**	**PCB 138 (95% CI)**	**PCB 153 (95% CI)**	**PCB 180 (95% CI)**	**Sum PCBs (95% CI)**
**% Change in thyroid function parameter according to 1% change in exposure**					
**Maternal TSH**					
BMI <19	**0.47 (0.06; 0.88)^#^**	**0.59 (0.18; 0.99)^#^**	**0.51 (0.15; 0.87)^#^**	**0.44 (0.1; 0.79)^#^**	**0.57 (0.18; 0.96)^#^**
BMI 19–25	0.04 (0.28; −0.20)	−0.05 (0.23; −0.32)	−0.09 (0.16; −0.34)	−0.12 (0.14; −0.37)	−0.067 (0.21; −0.35)
BMI ≥ 25	−0.18 (0.12; −0.48)	−0.13 (0.14; −0.41)	−0.15 (0.12; −0.42)	−0.17 (0.10; −0.45)	−0.16 (0.13; −0.45)
**Maternal FT4**					
BMI <19	0.11 (−0.02; 0.25)	0.09 (−0.04; 0.23)	0.08 (−0.04; 0.20)	0.08 (−0.03; 0.20)	0.1 (−0.03; 0.22)
BMI 19–25	(0.11; −0.08)	−0.04 (−0.14; 0.06)	0.01 (−0.09; 0.09)	0.04 (−0.03; 0.12)	0.01 (0.08; −0.07)
BMI ≥ 25	0.08 (0.19; −0.04)	0.01 (−0.09; 0.11)	0.03 (−0.07; 0.13)	0.05 (−0.03; 0.13)	0.04 (0.13; −0.05)
**Maternal TG Ab**					
BMI <19	0.37 (−0.22; 0.96)	0.17 (−0.41; 0.76)	0.12 (−0.40; 0.63)	0.08 (−0.42; 0.58)	0.15 (−0.41; 0.71)
BMI 19–25	−0.07 (−0.40; 0.27)	−0.03 (−0.41; 0.36)	0.08 (−0.29; 0.45)	0.20 (−0.18; 0.58)	0.07 (−0.32; 0.46)
BMI ≥ 25	0.13 (−0.30; 0.55)	0.14 (−0.27; 0.54)	0.08 (−0.30; 0.47)	0.12 (−0.28; 0.54)	0.12 (−0.31; 0.55)
**Maternal TPO Ab**					
BMI <19	**0.53 (0.04; 1.03)^#^**	0.48 (−0.02; 0.98)	**0.46 (0.02; 0.90)^#^**	0.32 (−0.11; 0.76)	0.48 (−0.004; 0.96)
BMI 19–25	−0.12 (−0.41; 0.17)	−0.08 (−0.41; 0.25)	−0.08 (−0.39; 0.23)	−0.03 (−0.37; 0.31)	−0.09 (−0.42; 0.25)
BMI ≥ 25	0.17 (−0.19; 0.54)	0.23 (−0.12; 0.57)	0.20 (−0.14; 0.53)	0.09 (−0.26; 0.44)	0.19 (−0.17; 0.56)
**Newborn total T4**					
BMI <19	0.02 (−0.19; 0.22)	−0.09 (−0.30; 0.12)	−0.10 (−0.10; 0.081)	−0.13 (−0.30; 0.052)	−0.10 (−0.30; 0.095)
BMI 19–25	−0.03 (0.07; −0. 14)	0.01 (0.15; −0.13)	0.01 (0.13; −0.12)	−0.02 (0.12; −0.16)	0 (0.13; −0.13)
BMI ≥ 25	−0.03 (0.11; −0.17)	−0.03 (0.12; −0.18)	−0.02 (0.10; −0.15)	−0.05 (0.10; −0.20)	−0.03 (0.11; −0.18)

§ *Mother age, newborn gender, PCBs levels, interaction between gender and PCB levels and interaction between BMI categories and PCB levels were included the model of linear regression*.

# *P < 0.05*.

*TSH, Thyroid-stimulating hormone; FT4, Free Thyroxine; Tg Ab, Thyroglobulin autoantibodies; TPO Ab, Thyroid peroxidase antibodies; NB T4, newborn total thyroxine; PCB, polychlorinated biphenyl*.

*Bold: p-value < 0.05*.

In multivariable analysis a 1% change in maternal ∑PCBs level was associated with a small, but statistically significant change in newborn total T4 levels of 0.13% (95% CI 0.04; 0.22) for males, but not females. For each specific PCB congener, the results showed the same trend (Table 7).

**Table 7:** Adjusted^§^ association between maternal PCB concentrations (lipid adj.) to total T4 in male newborns in the EHF-Assaf-Harofeh-Ichilov birth cohort, Israel, 2013–2015 (*n* = 85).

**PCB 118 (95% CI)**	**PCB 138 (95% CI)**	**PCB 153 (95% CI)**	**PCB 180 (95% CI)**	**Sum PCBs (95% CI)**
**% Change in thyroid function parameter according to 1% change in exposure**				
0.11 (0.03; 0.18)^#^	0.11 (0.03; 0.2)^#^	0.12 (0.03; 0.21)^#^	0.12 (0.04; 0.22)^#^	0.13 (0.04; 0.22)^#^

§ *Mother age, PCBs levels were included the model of linear regression*.

# *P < 0.05*.

*NB T4, newborn total thyroxine; PCB, polychlorinated biphenyl*.

After controlling for maternal age, PCB levels and BMI groups, the association between newborn sex and newborn total T4 was not statistically significant 0.001 95% CI (−0.07; 0.07). The interaction between maternal PCBs and maternal thyroid parameters was not statistically significantly different between males and females. In addition, there was no significant difference in all studied maternal and newborn thyroid parameters between males and females (data not shown).

## Discussion

In our birth cohort study of 157 mothers-newborns dyads, we aimed to evaluate the possible associations between background exposure to PCB congeners (118, 138, 153, and 180) at environmentally relevant levels and maternal and newborn thyroid hormones.

A significant positive correlation was found between maternal ∑PCBs and maternal TSH levels in women with pre-pregnancy BMI < 19. Maternal TPO Ab were highly correlated to congeners PCB 118 and 138, approaching significance to PCB153, but not to PCB180 or ∑PCBs in women with pre-pregnancy BMI < 19 (Table 4). No correlation was found in women with normal BMI (19–24.9). In women with BMI ≥ 25, the correlation tends to be negatively associated. In multivariable analysis the same trends were observed for maternal TSH and maternal TPO Ab. After adjustment, in women with pre-pregnancy BMI < 19 group, a 1% increase in the levels of ∑PCBs or either of the studied congeners was associated with a 0.44–0.57% increase in the maternal TSH levels and a 0.46–0.53% increase in maternal TPO Ab. The clinical relevance of this increase is ambiguous since the change is within the normal range, however, the public health relevance on a population level is much greater. Environmentally relevant maternal levels of PCBs are associated with an increase in maternal TPO Ab implying that clinical thyroid disease later in life in a significant proportion of women might be attributable to PCBs. In small observational studies, elevated TPOAb levels have been positively associated with exposure to PCBs (54, 55). PCBs might affect the development of autoimmune thyroid disease (56).

A possible explanation for these results is related to the rate of turnover of adipose tissue. Since PCBs are lipophilic, they have a higher affinity to fat tissue. Theoretically, women with low BMI have a more rapid turnover of PCBs, and a more significant effect is observed compared to women with high BMI (more fat tissue that acts as a “buffer,” slower turnover) (57). Body fat distribution is an important factor underlying metabolic abnormalities. Visceral adipose tissue differs from subcutaneous adipose tissue in its endocrine function, lipolytic activity, and immunologic function (58, 59). Straughen et al. found that visceral adipose tissue increased across pregnancy significantly more rapidly for normal weight compared with overweight/obese women (45).

In our cohort, women with higher BMI were older, but it was the only difference between the BMI groups. No other differences were observed between the groups (Table 1). There were no statistically significant differences between the 3 BMI groups regarding individual PCB levels or ∑PCBs level, maternal TSH, FT4, TPO Ab, and Tg Ab. However, the median TSH levels in women with BMI < 19 were higher −2.79 μIU/ml vs. 2.55 μIU/ml compared to women with BMI ≥ 25, with range (2.49–4.24) vs. (1.95–3.37), respectively. A study from Finland aimed to calculate gestational age-specific thyroid function and the associations between body mass index (BMI) and thyroid hormones, reported that the upper limits for TSH in women with BMI > 30 was higher than in women with BMI 20–25 (60). A study among Thai pregnant women found that BMI was a negative predictor of free T4, although there was no correlation between anti-TPO Abs and BMI (61). In our study, the association was found to be reversed, probably due to exposure to the studied PCBs.

We found no correlation between different PCB congeners or ∑PCBs concentrations in maternal serum and newborn's total T4 levels. Statistically significant correlation was found between PCBs and total T4 in male, but not in female newborns. However, after controlling for maternal age, PCB levels and BMI groups, the association between newborn sex and newborn total T4 was not statistically significant 0.001 95%CI (−0.07; 0.07).

Berg et al. similar to our study, have suggested that background exposure to POPs can alter pregnant women's thyroid function, but in opposite direction (62). They reported that among 19 POPs, eight different PCBs were detected in more than 80% of the samples, including the 4 PCBs tested in our cohort. Several individual PCBs were inversely associated with maternal T3, T4 and FT4, but not with infant TSH. A Canadian cohort study found that PCBs were negatively correlated with total maternal T3 in early pregnancy and with free T3 at delivery, but were not correlated with thyroxin levels (63). Polybrominated diphenyl ethers (PBDEs) and 3 PCBs were tested in this study, the same PCB congeners as in our cohort. A study performed in Michigan residents found a positive association between serum levels of the PCB 118, 138, 153, and 180 congeners and greater total and free T4 and total T3, which were stronger among women (19).

De Cock et al. found no association between cord plasma PCB 153 levels and total T4 in either male or female newborns (64). The Hokkaido birth cohort study (65) reported no significant association between maternal or neonatal TH and total PCBs or non-dioxin-like PCBs. However, they found associations between dioxin-like compounds and elevated neonatal FT4 levels, especially in males. Baba et al. measured multiple octachlorodibenzo-p-dioxin and dibenzofurans (OCDD/Fs) and PCBs, two of these PCBs were measured in our study. Later, the same group published another study examining PCB metabolites—hydroxylated PCBs (OH-PCBs) and maternal and neonatal thyroid hormones (17). They found that maternal exposure to OH-PCBs may increase maternal and neonatal FT4 levels, and a significant positive association was found between maternal ΣOH-PCB and male's FT4 levels, whereas no significant association was observed in female neonates.

While there is substantial evidence for the associations between exposure to PCBs and thyroid function in pregnant women, associations in neonates are more inconsistent. The heterogeneity observed in the studies could be related to differences in design, exposure levels, maternal age, the analytical method used, the period of pregnancy when thyroid hormones were analyzed, differences in the EDC compounds assessed, and the genetic variations. Llop et al. explored the role of genetic polymorphisms in DIO-encoding genes in the association between maternal organochlorine compounds (including PCBs 138, 153, and 180) and thyroid hormone levels (66), suggesting that the magnitude of the association between maternal organochlorine compounds exposure and thyroid parameters, depended on the genotype for the DIO1 gene (encoding iodothyronine deiodinases, important enzymes for the synthesis and determination of TH concentration).

In the last decades, epidemiological studies have reported endocrine and reproductive disorders with persistent organic pollutants (POPs) exposure during early life. The intrauterine period is exceptionally susceptible to chemical exposures and their effects on gene expression with epigenetic alteration such as DNA methylation and chromatin remodeling (67, 68). Mechanisms of PCB neurotoxicity are not fully understood and most probably involve effects on neurotransmitters, hormonal balance, and intracellular signaling (30, 69, 70). Some PCBs have structural similarity to thyroxine (T4), and these compounds can interfere with thyroid hormone production, receptor binding, or transport, resulting in altered hormone levels (71–73). Because thyroid hormones play a significant role in brain development (74), this may explain some of PCBs' neurodevelopmental effects (29, 75). Studies on animals have found allelic differences in the aryl hydrocarbon receptor, which affect sensitivity to PCB exposure, resulting in cognitive deficits and motor dysfunction. This data suggest that the AhR pathway plays a role in PCB neurotoxicity (22).

Maternal PCB serum levels measured in our cohort are consistent with the reduction in PCB levels found in the blood of Israeli donors over the last decades since 1975 (76). The same trend was proven for PCB levels and other contaminants in Israeli mothers' breast milk, with a significant reduction over the years (77, 78). This trend of reduction in contaminations in the environment is explained due to restrictions on Israeli agriculture and industry. PCB levels measured in our cohort are consistent with levels reported in other studies. The data from the MIREC study, a prospective birth cohort study of women from ten Canadian cities between 2008 and 2011, reported mean ∑PCBs (118, 138, 153, 170, 180, 187) concentration of 34.9 ng/g lipids (79).

Our study has several strengths and limitations. Assessment of potential thyroid impairment is difficult due to the thyroid system's complexity, especially during pregnancy. Therefore, we included all significant components in the maternal thyroid homeostasis. As for newborn assessment—we had only the total T4 results without TSH levels from the National survey screening. Total T4 screening may be enough for hypothyroidism screening but might be insufficient to assess the effect of PCBs exposure. A recent study combined three European mother–child cohorts and found an association between exposure to PCB-153, p,p′-DDE and newborn TSH levels (20).

Since pregnancy-specific TSH reference range is not available in our medical center, we used an upper reference limit of ~4.0 μIU/ml, based on the recent recommendation of the American Thyroid Association (ATA). About 20% of all participating women had values above this pregnancy-specific upper limit. For these pregnant women at high risk for thyroid disease the ATA recommends TSH levels be measured with a reflex anti-TPO antibody if TSH is 2.5–10 μIU/ml (80). In our cohort, only three women were TPO Ab positive, two of them had TSH levels above 2.5 μIU/ml, and only one was Tg Ab positive.

PCBs are a large family of 209 congeners, our study tested only 4 congeners. However, PCBs 118, 138, 153, 180, are among the most frequently detected congeners in white adipose tissue and blood samples (9, 10). These PCBs have the highest detection frequencies in the US population and have a half-life of years.

It has been recommended that regression modeling be carried out both with and without lipid correction for lipophilic compounds in the blood (50), but it has rarely been followed, and lipid-based models are commonly used. PCBs are typically associated with the lipid matrix in tissues because of their non-polar and lipophilic character, and the results may be expressed per weight of lipid rather than on a whole weight or a volume basis. If equilibrium is reached, information regarding serum PCB levels and serum lipid levels may be predictive of PCB body burden (81). Total lipids were calculated for the mothers and no statistically significant differences were observed between the three BMI groups (*p* = 0.06). Moreover, we performed the correlation analysis between the wet-weighted PCBs (not lipid adjusted) to the thyroid parameters of mother and newborn. The same trends as in lipid-adjusted correlations were observed (Supplementary Tables 2, 3). There are physiological variations in lipid metabolism during pregnancy, with a marked increase of lipids levels during the third trimester of pregnancy (82). Considering the changes in lipid metabolism during pregnancy, the use of a lipid-adjusted model is appropriate in our setup.

## Conclusions

The present study may suggest that background low-dose exposure to some PCBs can alter thyroid hormone homeostasis in pregnant women, and might be associated with abnormal TSH levels and TPO-Ab in women with low BMI. However, in women with high BMI (>25), this trend is inverse.

Forthcoming, the remaining samples of recruited mother-father-infant triads will be studied including blood, urine, cord blood, breast milk and meconium, for concentrations of PCBs and other contaminants. Additionally, our cohort of children is being followed prospectively for future studies to evaluate developmental outcomes.

## Data Availability Statement

The datasets presented in this study can be found in online repositories. The names of the repository/repositories and accession number(s) can be found below: https://ipchem.jrc.ec.europa.eu/#discovery.

## Ethics Statement

The studies involving human participants were reviewed and approved by Helsinki committee, Shamir Medical Center (Assaf Harofeh). Written informed consent to participate in this study was provided by the participants' legal guardian/next of kin. Written informed consent was obtained from the individual(s), and minor(s)' legal guardian/next of kin, for the publication of any potentially identifiable images or data included in this article.

## Author Contributions

MBer, DB, AB, EK, AL, RKe, JT, MBet, MM, AO, PF-L, MBr, TZ-B, MBerk, IM, and RM: substantial contributions to the conception or design of the work, or the acquisition, analysis, or interpretation of data for the work. MBer, DB, DM, RL, PF-L, TZ-B, RKo, CK, MBerk, IM, and RM: drafting the work or revising it critically for important intellectual content. MBer, MBerk, and RM: agree to be accountable for all aspects of the work in ensuring that questions related to the accuracy or integrity of any part of the work are appropriately investigated and resolved. All authors contributed to the article and approved the submitted version.

## Funding

This work was supported by the Environment and Health Fund (EHF)-Grant No. RGA1202.

## Conflict of Interest

The authors declare that the research was conducted in the absence of any commercial or financial relationships that could be construed as a potential conflict of interest.

## Publisher's Note

All claims expressed in this article are solely those of the authors and do not necessarily represent those of their affiliated organizations, or those of the publisher, the editors and the reviewers. Any product that may be evaluated in this article, or claim that may be made by its manufacturer, is not guaranteed or endorsed by the publisher.
