# Supplementary material for: Maternal and Newborn Thyroid Hormone, and the Association With Polychlorinated Biphenyls (PCBs) Burden: The EHF (Environmental Health Fund) Birth Cohort
Source: Front Pediatr. 2021 Sep 13;9:705395. doi: 10.3389/fped.2021.705395 (PMC8473683; doi:10.3389/fped.2021.705395)
Supplement: Supplementary file 1 [file Table_1.DOCX]

Supplementary Material

**Table 1**

**Demographic characteristics of mothers in the EHF-Assaf-Harofeh-Ichilov birth cohort, Israel, 2013-2015, comparing those included (n = 157) with those excluded (n = 106).**

| **Maternal characteristics** | **Included** | **Excluded** | **P value** | |
| --- | --- | --- | --- | --- |
| **Number** | **157** | **106** |  | |
| Maternal age at delivery (years)  Mean ± SD | 31.9 ± 4.8 | 32.8 ± 4.4 | 0.131 | |
| **Parity** |  |  |  | |
| primiparous (yes) | 50 (31.8%) | 20 (18.9%) | **0.015** | |
| Years of education (median, IQR) | 16  (13-17) | 15.5  (13.25-17) | 0.759 | |
| Stable relationship (yes) (N, %) | 152 (96.8%) | 98 (98%) | 0.709 | |
| Employed (yes) (N, %) | 138 (87.9%) | 93 (93%) | 0.186 | |
| Has Ever Smoked During Pregnancy (yes) (N, %) | 11 (7.0%) | 8 (8.5%) | 0.75 | |
| Has Ever Consumed Alcohol During Pregnancy (yes) (N, %) | 13 (8.3%) | 9 (9.2%) | 0.815 | |
| **Monthly Household Income (Net)** |  |  | 0.815 | |
| Less than 17,000 NIS (yes) (N, %) | 106 (67.5%) | 64 (71.9%) |  | |
| 17,001 NIS and more (yes) (N, %) | 38 (24.2%) | 25 (28.1%) |  | |
| **Country of origin** |  |  | **0.022** | |
| Israel (N, %) | 133 (84.7%) | 73 (73%) |  | |
| Other (N, %) | 24 (15.3%) | 27 (27%) |  | |
| Pre-pregnancy BMI (median, IQR) | 21.1  (19.3-22.9) | 22.5  (20.5-24.3) | **0.008** | |
| **Bold** - parameters with statistically significant difference, p<0.05.  IQR - interquartile range; SD - Standard Deviation.  Kruskal Wallis, Pearson Chi-square or Fisher's exact tests were used. | | | |  |

**Table 2**

**Spearman rank correlations of PCBs (wet-weighted) and thyroid function of mothers and newborns in the EHF-Assaf-Harofeh-Ichilov birth cohort, Israel, 2013-2015 (n = 157)**

|  | | **Maternal TSH** | **Maternal FT4** | **Maternal**  **Tg Ab** | **Maternal**  **TPO Ab** | **NB T4** |
| --- | --- | --- | --- | --- | --- | --- |
| PCB 118 (pg/ml)  **r**  **p** | | .047 | -.007 | .049 | .072 | .132 |
|  |  | .558 | .927 | .543 | .373 | .099 |
| PCB 138 (pg/ml)  **r**  **p** | | .016 | -.020 | .104 | .123 | .115 |
|  |  | .844 | .808 | .197 | .125 | .150 |
| PCB 153 (pg/ml)  **r**  **p** | | .022 | .015 | .103 | .131 | .103 |
|  |  | .783 | .852 | .198 | .102 | .198 |
| PCB 180 (pg/ml) **r**  **p** | | .009 | .022 | .083 | .094 | .059 |
|  |  | .915 | .787 | .301 | .241 | .465 |
| Sum PCBs (pg/ml) **r**  **p** | | .025 | .011 | .093 | .116 | .100 |
|  |  | .753 | .893 | .248 | .149 | .214 |
| **Male (N=85)** | | | | | | |
| PCB 118 (pg/ml)  **r**  **p** | | .002 | .025 | .113 | .043 | **.245** |
|  |  | .986 | .817 | .303 | .694 | **.024#** |
| PCB 138 (pg/ml)  **r**  **p** | | -.048 | .074 | .163 | .051 | **.251** |
|  |  | .664 | .502 | .135 | .643 | **.020#** |
| PCB 153 (pg/ml)  **r**  **p** | | -.017 | .087 | .183 | .092 | **.237** |
|  |  | .874 | .427 | .093 | .400 | **.029#** |
| PCB 180 (pg/ml)  **r**  **p** | | -.035 | .101 | .176 | .092 | **.223** |
|  |  | .753 | .356 | .107 | .404 | **.040#** |
| Sum PCBs (pg/ml) **r**  **p** | | -.020 | .090 | .174 | .078 | **.242** |
|  |  | .859 | .412 | .111 | .477 | **.026#** |
| **Female (N=72)** | | | | | | |
| PCB 118 (pg/ml)  **r**  **p** | | .083 | -.014 | -.015 | .092 | -.041 |
|  |  | .490 | .906 | .902 | .441 | .734 |
| PCB 138 (pg/ml)  **r**  **p** | | .099 | -.112 | .049 | .210 | -.069 |
|  |  | .408 | .348 | .684 | .077 | .563 |
| PCB 153 (pg/ml)  **r**  **p** | | .071 | -.039 | .034 | .184 | -.077 |
|  |  | .552 | .746 | .779 | .123 | .520 |
| PCB 180 (pg/ml) **r**  **p** | | .054 | -.056 | .011 | .104 | -.146 |
|  |  | .655 | .641 | .929 | .383 | .222 |
| Sum PCBs (pg/ml)  **r**  **p** | | .080 | -.045 | .029 | .164 | -.086 |
|  |  | .505 | .705 | .808 | .169 | .471 |
| **r** – Spearman's rho | | | | | | |
| **p** – p-value  # P value <0.05 | | | | | | |

**Table 3**

**Spearman rank correlations of PCBs (wet-weighted) and thyroid function of mothers and newborns at different BMI groups**, **EHF-Assaf-Harofeh-Ichilov birth cohort, Israel, 2013-2015, grouped by maternal pre-pregnancy BMI.**

|  | **Maternal TSH** | **Maternal FT4** | **Maternal**  **Tg Ab** | **Maternal**  **TPO Ab** | **NB T4** |
| --- | --- | --- | --- | --- | --- |
| **BMI<19 (N=24)** | | | | | |
| PCB 118 (pg/ml) **r**  **p** | **.502** | .171 | .250 | **.530** | -.162 |
|  | **.013#** | .423 | .239 | **.008#** | .448 |
| PCB 138 (pg/ml) **r**  **p** | **.616** | .276 | .196 | **.437** | -.068 |
|  | **.001#** | .192 | .359 | **.033#** | .752 |
| PCB 153 (pg/ml) **r**  **p** | **.579** | .225 | .188 | **.498** | -.134 |
|  | **.003#** | .290 | .378 | **.013#** | .534 |
| PCB 180 (pg/ml) **r**  **p** | **.548** | .123 | .044 | .296 | -.144 |
|  | **.006#** | .566 | .838 | .160 | .502 |
| Sum PCBs (pg/ml) **r**  **p** | **.562** | .232 | .166 | **.476** | -.180 |
|  | **.004#** | .274 | .439 | **.019#** | .400 |
| **BMI 19-25 (N=88)** | | | | | |
| PCB 118 (pg/ml) **r**  **p** | .070 | -.133 | -.056 | -.129 | .132 |
|  | .518 | .218 | .605 | .230 | .219 |
| PCB 138 (pg/ml) **r**  **p** | .028 | -.143 | .018 | -.046 | .149 |
|  | .797 | .182 | .865 | .669 | .165 |
| PCB 153 (pg/ml) **r**  **p** | .025 | -.113 | .047 | -.025 | .111 |
|  | .817 | .295 | .667 | .818 | .303 |
| PCB 180 (pg/ml) **r**  **p** | .011 | -.076 | .052 | -.028 | .049 |
|  | .919 | .479 | .632 | .793 | .650 |
| Sum PCBs (pg/ml) **r**  **p** | .034 | -.121 | .026 | -.047 | .116 |
|  | .751 | .263 | .811 | .665 | .281 |
| **BMI ≥25 (N=45)** | | | | | |
| PCB 118 (pg/ml) **r**  **p** | -.171 | .164 | .192 | .254 | **.318** |
|  | .262 | .282 | .208 | .092 | **.033#** |
| PCB 138 (pg/ml) **r**  **p** | -.244 | .071 | .193 | .245 | .178 |
|  | .106 | .644 | .204 | .105 | .243 |
| PCB 153 (pg/ml) **r**  **p** | -.248 | .087 | .145 | .212 | .217 |
|  | .100 | .570 | .344 | .161 | .153 |
| PCB 180 (pg/ml) **r**  **p** | -.265 | .102 | .109 | .156 | .176 |
|  | .078 | .507 | .476 | .305 | .246 |
| Sum PCBs (pg/ml) **r**  **p** | -.256 | .082 | .158 | .206 | .199 |
|  | .090 | .592 | .301 | .174 | .190 |
| **r** – Spearman's rho | | | | | |
| **p** – p-value  # P value <0.05 | | | | | |
